# Antiarthritic and Antinociceptive Properties of Ylang-Ylang (*Cananga odorata*) Essential Oil in Experimental Models

**DOI:** 10.3390/cimb46080534

**Published:** 2024-08-18

**Authors:** Paloma Kênia de Moraes Berenguel Lossavaro, Josyelen Lousada Felipe, Joyce dos Santos Lencina, Iluska Senna Bonfá, Kamylla Fernanda Souza de Souza, Lucas Luiz Machado, Mila Marluce Lima Fernandes, João Victor Ferreira, Maria Inês Lenz Souza, Luciane Candeloro, Cândida Aparecida Leite Kassuya, Edgar Julian Paredes-Gamero, Eduardo Benedetti Parisotto, Mônica Cristina Toffoli-Kadri, Saulo Euclides Silva-Filho

**Affiliations:** 1 Pharmaceutical Sciences, Food and Nutrition College, Federal University of Mato Grosso do Sul, Campo Grande 79070-900, MS, Brazil; paloma.lossavaro01@gmail.com (P.K.d.M.B.L.); josyfelipe@hotmail.com (J.L.F.); joycedslencina@gmail.com (J.d.S.L.); iluska.senna@ufms.br (I.S.B.); kamylla.bio@outlook.com (K.F.S.d.S.); lucaslmachado11@gmail.com (L.L.M.); mila.fernandes@ufms.br (M.M.L.F.); joao_ferreira@ufms.br (J.V.F.); edgar.gamero@ufms.br (E.J.P.-G.); eduardo.parisotto@ufms.br (E.B.P.); monica.kadri@ufms.br (M.C.T.-K.); 2 Biosciences Institute, Federal University of Mato Grosso do Sul, Campo Grande 79070-900, MS, Brazil; maria.souza@ufms.br (M.I.L.S.); lucandeloro@gmail.com (L.C.); 3 Health Sciences College, Federal University of Grande Dourados, Dourados 79804-970, MS, Brazil; candida2005@gmail.com

**Keywords:** essential oil, ylang-ylang, arthritis, persistent inflammation, natural products

## Abstract

The aim of this study was to evaluate the effect of ylang-ylang (*Cananga odorata*) essential oil (YEO) on models of experimental arthritis, persistent inflammation, and nociception in mice. YEO treatment at doses of 100 and 200 mg/kg reduced the infiltration of leukocytes into the joint cavities of mice submitted to zymosan-induced arthritis 6 h and 7 days after arthritis induction. At these doses, YEO treatment reduced the formation of joint edema 4 and 6 h after arthritis induction, and at a dose of 200 mg/kg, YEO treatment reduced mechanical hyperalgesia 3 and 4 h after arthritis induction. At the dose of 200 mg/kg, YEO treatment reduced interleukin-6 (IL-6) levels and cartilage destruction in the zymosan-induced arthritis model, and reduced edema formation and mechanical hyperalgesia in the model of persistent inflammation (21 days) induced by complete Freund’s adjuvant (CFA) in mice. YEO treatment at a dose of 200 mg/kg reduced the nociceptive response in experimental models of nociception induced by acetic acid and formalin. The YEO treatment reduced inflammatory parameters in the experimental arthritis model, and presented antiarthritic, anti-hyperalgesic, antinociceptive, and anti-inflammatory properties.

## 1. Introduction

Inflammation is a biological response against injury or a pathogen; this response promotes clinical signs such as heat, redness, pain, swelling, and loss of tissue function [1], and it can be divided into two phases: acute or chronic. Acute inflammation has a short duration and involves neutrophils as the predominant leukocyte and the first cells recruited to the inflammatory site, in addition to vasodilation, increased vascular permeability, and the extravasation of plasma proteins [2,3]. Chronic inflammation develops over weeks or years and is marked by tissue destruction and the infiltration of mononuclear cells, such as monocytes and lymphocytes, in addition to fibrosis and angiogenesis [4,5]. This process results from the persistence of the inflammatory stimulus or the ineffectiveness of the acute inflammatory response, which can generate chronic or autoimmune pathological processes, such as Alzheimer’s disease, multiple sclerosis, and rheumatoid arthritis (RA) [5].

RA is an autoimmune disease that causes chronic inflammation of the joints. Several symptoms are described for RA, such as swelling and joint pain, progressive joint deformity, synovial membrane destruction, and remodeling. It is a multifactorial disease, and mainly affects women [6]. Patients with RA have a reduced life expectancy due to morbidity and complications resulting from the disease. RA treatment involves many drug classes, such as non-steroidal anti-inflammatory drugs (NSAIDs), corticosteroids, and disease-modifying antirheumatic drugs (DMARDs) [7]. Hormonal or non-hormonal anti-inflammatory drugs are drugs used to treat acute or chronic inflammatory diseases; however, the prolonged use of these drugs is associated with adverse effects, such as renal, hepatic, and cardiovascular toxicity [6]. Therefore, aromatic plants and natural products with anti-inflammatory properties have been used for years by the population as a therapeutic alternative for the most diverse diseases [8].

*Cananga odorata* Hook. F. & Thomson, popularly known as ylang-ylang, is an aromatic plant. This species is native to Asia and can be found in humid tropical countries [9]. Ylang-ylang essential oil (YEO) is obtained from *C. odorata* flowers and is used in cosmetics and aromatherapy [9,10]. Several constituents of YEO, such as alcohols, esters, ketones, and aliphatic aldehydes, are responsible for fragrance [9,11]. Some beneficial biological properties of YEO are described in the literature, such as antimicrobial [12], antioxidant [13], anti-inflammatory [14], and antirheumatic [9,15,16] activities. And recently, YEO has been shown to be capable of relieving neuropathic pain and improving pain-associated anxiety [17]. The toxicity of YEO in mice is not considered to be high (LD_50_ > 2 g/kg) [14].

A previous study, carried out by our research group, demonstrated that treatment with YEO reduced the formation of paw edema, mechanical hyperalgesia, and leukocyte recruitment during the acute inflammatory response in vivo, in addition to reducing chemotaxis and the phagocytic activity of neutrophils in vitro [14]. However, there are no studies demonstrating YEO’s activity in experimental models of arthritis and persistent inflammation.

Therefore, due to the limited use of anti-inflammatory drugs, research on natural products with anti-inflammatory activity and less harmful effects is relevant. Thus, this work aimed to evaluate the effect of YEO on an experimental arthritis model, as well as to evaluate the effect of this essential oil on persistent inflammation and nociception.

## 2. Materials and Methods

### 2.1. Chemicals and YEO Obtention

YEO, zymosan, complete Freund’s adjuvant (CFA), indomethacin, dexamethasone, and morphine were obtained commercially from Sigma-Aldrich^®^ (St. Louis, MO, USA). All other chemicals used were analytical grade.

The YEO’s phytochemical composition was previously described in a study carried by our group, in which we observed the presence of benzyl acetate (18.21%), linalool (15.23%), benzyl benzoate (11.39%), geranyl acetate (9.46%), methyl benzoate (7.64%), *p*-methyl anisole (7.38%), *trans*-caryophyllene (5.42%), germacrene D (4.61%), and benzyl salicylate (4.47%) [14].

### 2.2. Animals

Male Swiss mice (weighing between 25 and 30 g) were provided by the Central Animal House of the Federal University of Mato Grosso do Sul (UFMS). The animals were housed at 22 ± 2 °C under a 12/12 h light/dark cycle. Before the experiments, the animals were fasted overnight, with water provided ad libitum. The experimental protocols were approved by the Ethical Committee in Animal Experimentation of the UFMS (protocol number: 1.182/2021).

### 2.3. Zymosan-Induced Arthritis Model

Male Swiss mice (n = 5–7 animals/group) were intra-articularly injected with 10 µL of zymosan solution (200 μg/knee joint). Zymosan was injected in the right knee joint of the animals, and saline solution 0.9% was injected into a naive group at the same volume. One hour before zymosan injection, the animals were treated with YEO (50, 100, and 200 mg/kg), dexamethasone (1 mg/kg, reference drug), or a vehicle (saline 0.9% containing 1% of Tween 80) via the oral route. Six hours or seven days after zymosan-induced arthritis, the mice were euthanatized. For the 7-day analyses, i.e., for the animals that were euthanized after 7 days of arthritis induction, the mice were treated with YEO, dexamethasone, or the vehicle 1 h before the zymosan intra-articular injection, as well as daily, once a day, for 7 days.

### 2.4. Knee-Joint Edema and Mechanical Hyperalgesia in Animals Submitted to Zymosan-Induced Arthritis

Mechanical hyperalgesia was evaluated 3 and 4 h after arthritis induction. The evaluation of mechanical hyperalgesia was performed after the animals were placed in a containment box with support for the analgesiometer test. The animals were allocated 30 min to adapt in order to decrease any exploratory behavior and support their four paws on the base. To measure the nociceptive mechanical sensitivity threshold of the joint, a digital analgesiometer (Von Frey, Insight^®^, Ribeirão Preto, SP, Brazil) [18] was used as a pressure transducer, which records the applied force (g) until the moment of paw withdrawal. The knee-joint edema formation was evaluated 4 and 6 h after arthritis induction. The evolution of edema formation was measured using a micrometer, which measured the edema formation in millimeters (mm). The evaluation of knee-joint edema formation was made by comparing the difference between the right and left knee joints.

### 2.5. Analysis of Leukocyte Infiltration into Articular Cavity of Animals Submitted to Zymosan-Induced Arthritis

Six hours or seven days after the arthritis induction, the mice were euthanized, and the knee joint was exposed by surgical incision and washed twice with 5 µL of phosphate-buffered saline (PBS) that contained ethylenediaminetetraacetic acid (EDTA). The supernatant was diluted to a final volume of 50 µL with PBS/EDTA to determine the total cell counts. The total number of leukocytes, measured using diluted Turk’s solution, was determined in a Neubauer chamber under a light microscope. The results were recorded as the number of leukocytes per cavity.

### 2.6. Determination of Interleukin-6 Levels in Articular Cavity of Animals Submitted to Zymosan-Induced Arthritis

Interleukin-6 (IL-6) dosage was performed on samples of femur–tibial joints collected 6 h after zymosan injection. The knee joints were processed and centrifuged, and the supernatants were collected. IL-6 levels in the supernatants were determined using ELISA kits. These data are expressed as picograms (pg)/mL.

### 2.7. Histological Analysis

Histological analysis was performed 7 days after the onset of zymosan-induced arthritis. The right knee joints were subsequently demineralized in a 10% formaldehyde solution for 3 days, and the formaldehyde was then replaced with EDTA dissolved in saline (3 g EDTA/30 mL). The joints were stored in 70% ethanol. The samples were then subjected to a dehydration process using an increasing series of ethanol (70, 80, 90, and 100 GL) diaphanized in xylol, and embedded in paraffin. The samples were then serially sectioned (at 4 µm thicknesses) using a rotary microtome (Leica RM2245). All sections were stained with Harris’s hematoxylin and eosin (H&E) and examined under a microscope (Olympus BX41; original magnification: 400×). The histological sections from each group were examined using a gradient scale of 0–2 according to the cartilage degradation (Table 1).

### 2.8. Model of Persistent Inflammation Induced by Complete Freund’s Adjuvant (CFA)

The persistent edema and mechanical hyperalgesia model induced by complete Freund’s adjuvant (CFA) was employed to study the analgesic and anti-inflammatory properties of prolonged treatment with YEO. At time zero, 20 μL of CFA (a suspension of killed Mycobacterium tuberculosis in oil) was injected into the right hind paw of the mice (intraplantar injection), and 20 μL of saline was injected into the left hind paw, while mice in the naive group had 20 μL of saline injected into both paws. The animals were treated with YEO (200 mg/kg), dexamethasone, or the vehicle (n = 5–7 animals/group) via the oral route 24 h after the injection of CFA, and for 21 days, once a day. The mechanical hyperalgesia and paw edema were measured 6, 11, 16, and 21 days after the CFA injection. The evaluation of mechanical hyperalgesia was performed using a digital analgesiometer (Von Frey, Insight^®^), as described earlier. The edema formation was measured with a plethysmometer (Insight^®^).

### 2.9. Acetic Acid-Induced Abdominal Writhing Model

The animals were treated with YEO (200 mg/kg), indomethacin (15 mg/kg), or the vehicle (n = 5–7 animals/group) 60 min before an injection of 0.6% (*v*/*v*) acetic acid solution, which was diluted in distilled water and administered intraperitoneally (i.p.) in a volume 10 mL/kg. The number of abdominal contortions presented by the animals was recorded 30 min (min) after the stimulus injection. Results were recorded as the number of writhing contortions.

### 2.10. Formalin-Induced Nociception Test

The method utilized in this assay was previously described in [20]. The animals were treated orally with the vehicle, indomethacin (15 mg/kg), morphine (5 mg/kg), or YEO (200 mg/Kg) (n = 5–7 animals/group). Thirty minutes after treatment for the morphine group and sixty minutes after treatment for the other groups, the animals were given a 40 µL intraplantar injection of 1.2% formalin, diluted in saline solution, into their right hind paw. The time that each animal spent licking or biting its paws was measured during the first (0–5 min) and the second (15–30 min) phases of the test. The results were recorded in seconds.

### 2.11. Statistical Analysis

Data are expressed as the mean ± SEM for each experimental group. The results were statistically analyzed by using a one-way variance analysis (ANOVA), followed by the Newman–Keuls test. The percentage of inhibition was calculated in relation to the control group. Differences were considered significant when *p* < 0.05.

## 3. Results

### 3.1. YEO Treatment Reduces Knee-Joint Edema Formation and Mechanical Hyperalgesia Induced by Zymosan

**Figure 1:**
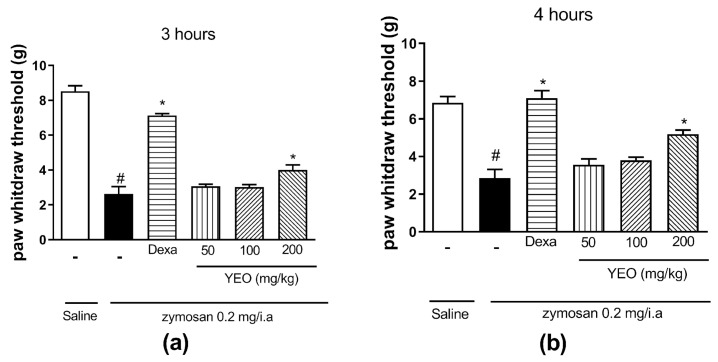
Effect of YEO treatment on mechanical hyperalgesia in Swiss mice submitted to zymosan-induced arthritis. This figure shows the values observed 3 h (**a**) and 4 h (**b**) after arthritis induction in the control (vehicle, p.o.), YEO (50, 100, and 200 mg/kg, p.o.), and dexamethasone (1 mg/kg, p.o.) groups. Results are expressed as the mean ± SEM. ^#^ *p* < 0.05 compared to the saline group (vehicle); * *p* < 0.05 compared to the control group (ANOVA, Newman–Keuls test).

The zymosan injection into the knee cavity of mice induced mechanical hyperalgesia at 3 and 4 h, compared to the saline group (Figure 1). At the 3 h time point after arthritis induction, the YEO treatment at a dose of 200 mg/kg reduced mechanical hyperalgesia in 52.38% of the mice (Figure 1a), compared to the control group. At the 4 h time point after arthritis induction, the YEO treatment at a dose of 200 mg/kg reduced mechanical hyperalgesia in 81.75% of the mice (Figure 1b), compared to the control group. Dexamethasone (reference drug) treatment also reduced mechanical hyperalgesia at the time points of 3 and 4 h after arthritis induction compared to the control group. YEO treatment at doses of 50 and 100 mg/kg did not significantly reduce the mechanical hyperalgesia at any tested time.

**Figure 2:**
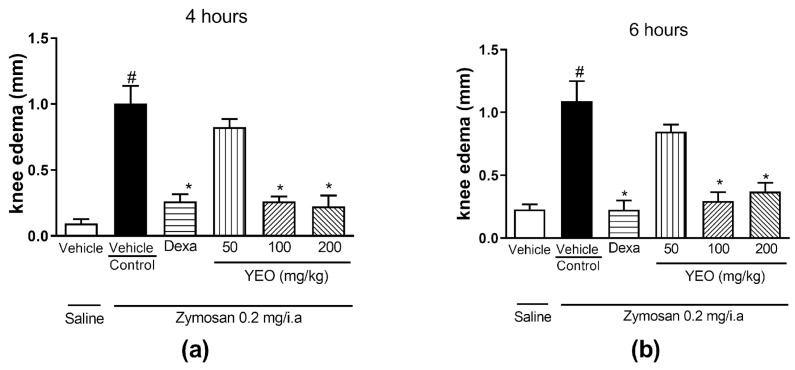
Effect of YEO treatment on edema formation in Swiss mice submitted to zymosan-induced arthritis. This figure shows the degree of knee edema formation at 4 (**a**) and 6 (**b**) hours after arthritis induction in the control (vehicle, p.o.), YEO (50, 100, and 200 mg/kg, p.o.), and dexamethasone (1 mg/kg, p.o.) groups. Results are expressed as the mean ± SEM. ^#^ *p* < 0.05 compared to the saline group (vehicle); * *p* < 0.05 compared to the control group (ANOVA, Newman–Keuls test).

The zymosan injection into the knee cavity of the mice induced the formation of edema at 4 h (1.00 ± 0.13 mm, Figure 2a) and 6 h (1.08 ± 0.16 mm, Figure 2b) after arthritis induction, compared to the group that received the saline solution (0.09 ± 0.03 mm and 0.23 ± 0.03 mm, 4 and 6 h after arthritis induction, respectively). YEO treatment showed anti-inflammatory activity, promoting a significant reduction in knee edema formation at doses of 100 and 200 mg/kg (Figure 2). YEO treatment at the dose of 100 mg/kg promoted a reduction in edema formation at all tested times, inhibiting the edema formation at 4 (Figure 2a) and 6 h (Figure 2b) in 73.91 and 72.89% of the animals, respectively, compared to the control group. At the dose of 200 mg/kg, YEO treatment promoted a reduction in edema formation at all times after zymosan injection, reducing the edema formation at 4 (Figure 2a) and 6 h (Figure 2b) in 77.5 and 66% of the animals, respectively, compared to the control group. The dexamethasone treatment promoted a reduction in knee edema formation at all time points tested (Figure 2a,b). YEO treatment at a dose of 50 mg/kg did not significantly reduce the edema formation at any of the tested times.

### 3.2. YEO Treatment Reduces Leukocyte Recruitment in a Zymosan-Induced Arthritis Model

**Figure 3:**
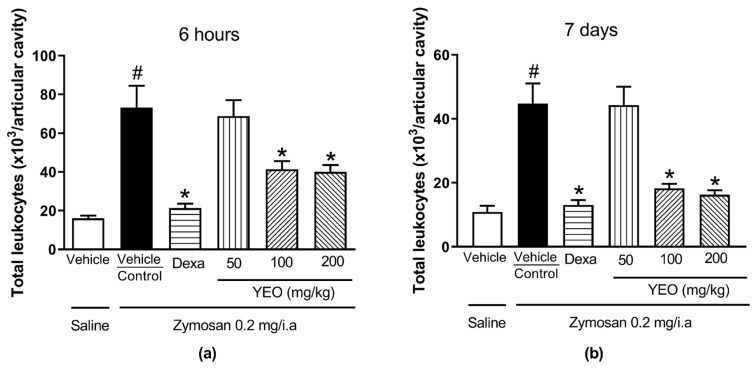
Effect of YEO treatment on migrated leukocyte number in the knee-joint cavities of Swiss mice 6 h (**a**) and 7 days (**b**) after zymosan-induced arthritis in the control (vehicle, p.o.), YEO (50, 100, and 200 mg/kg, p.o.), and dexamethasone (1 mg/kg, p.o.) groups. ^#^ *p* < 0.05 compared to saline treatment (vehicle); * *p* < 0.05 compared to the control group (ANOVA, Newman–Keul test).

Six hours after arthritis induction, we observed an increase in the number of leukocytes in the mice’s knee-joint cavities (73.25 ± 11.24 × 10^3^ cells/cavity) compared with the group that received only saline injections (16.00 ± 1.47 × 10^3^ cells/cavity). YEO treatment at the doses of 100 and 200 mg/kg significantly reduced the number of total leukocytes (43.68% and 45.39%, respectively) in the knee-joint cavities of mice in the arthritis group (Figure 3a). Seven days after arthritis induction, we also observed an increase in the number of leukocytes in the mice’s knee-joint cavities (44.75 ± 6.32 × 10^3^ cells/cavity) compared with the group that received only saline injections (10.80 ± 2.03 × 10^3^ cells/cavity). YEO treatment at the doses of 100 and 200 mg/kg significantly reduced the number of total leukocytes (59.21% and 63.68%, respectively) in the mice’s knee-joint cavities (Figure 3b).

### 3.3. YEO Treatment Reduces IL-6 Levels in a Zymosan-Induced Arthritis Model

**Figure 4:**
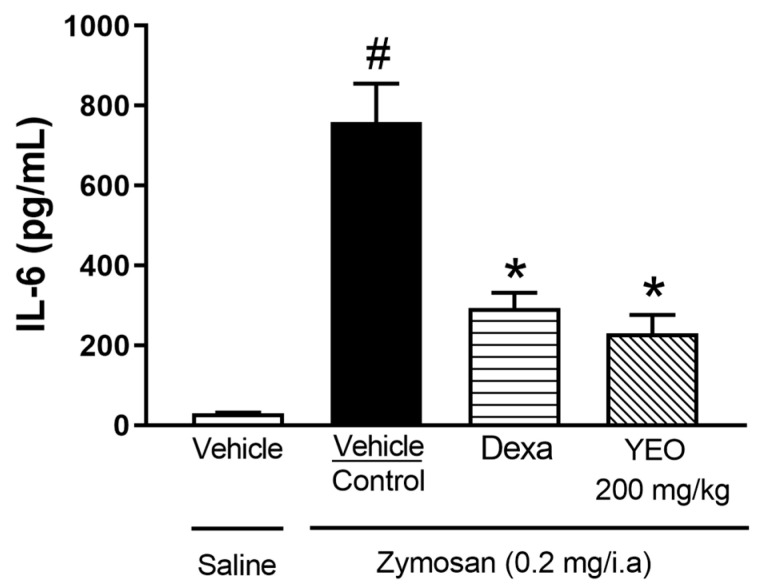
Effect of YEO treatment on IL-6 levels in the synovial fluid of Swiss mice 6 h after zymosan-induced arthritis in the control (vehicle, p.o.), YEO (200 mg/kg, p.o.), and dexamethasone (1 mg/kg, p.o.) groups. ^#^ *p* < 0.05 compared to saline treatment (vehicle); * *p* < 0.05 compared to the control group (ANOVA, Newman–Keul test).

Synovial fluid samples collected 6 h after zymosan-induced arthritis were subjected to ELISA assays for determining their levels of IL-6. Our results demonstrated that the IL-6 levels were increased in animals that received zymosan injections, compared to the animals that received saline injections. The YEO treatment, at a dose of 200 mg/kg, reduced the IL-6 levels in 69.67% of the animals. The dexamethasone (reference drug) reduced the IL-6 levels in 61.33% (Figure 4).

### 3.4. YEO Reduces Cartilage Damage in Mice Submitted to Zymosan-Induced Arthritis

**Figure 5:**
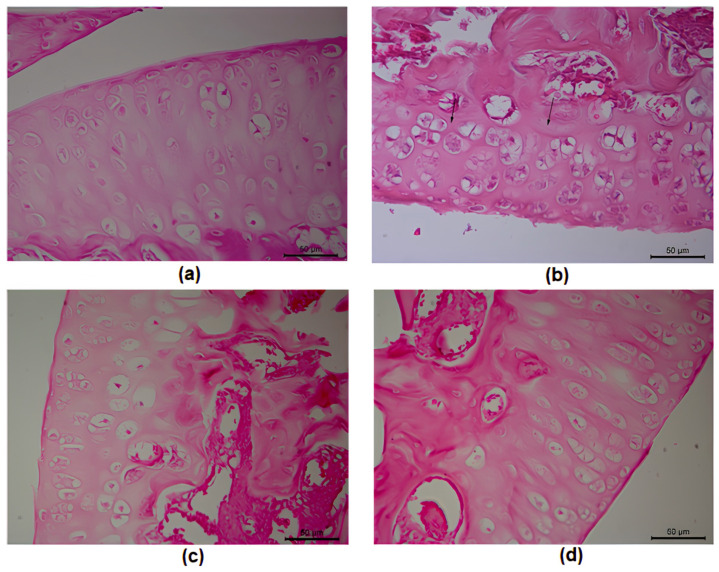
Cartilage histopathology degradation assessment in the knee-joint cavities of *Swiss* mice 7 days after zymosan-induced arthritis: results of saline treatment (vehicle) (**a**); control treatment (vehicle, p.o.), showing fibrillation (arrows) (**b**); YEO treatment (200 mg/kg, p.o.) (**c**); and dexamethasone treatment (1 mg/kg, p.o.) (**d**). These images demonstrate that discontinuity on the surface of the synovial membrane, fibrillation (arrows), disorientation of the chondrocyte columns, cell death, proliferation (clustering), and hypertrophy were observed in the arthritic animals when compared to the animals that received only saline injections.

In the histological analysis, discontinuity on the surface of the synovial membrane, fibrillation (see arrows in Figure 5), disorientation of the chondrocyte columns, cell death, proliferation (clusters), and hypertrophy (Figure 5b) were observed in the arthritic animals, compared to the animals that received only saline injections (Figure 5a), where we observed the surface and cartilage morphology to be intact. Furthermore, in the group treated with YEO (200 mg/kg) and dexamethasone for 7 days, we noted a reduction in cartilage damage to grade 1 (Figure 5c,d), where the cartilaginous surface was intact with a homogeneous matrix, despite proliferation (clusters) and chondrocyte hypertrophy still being observed. In the dexamethasone group, we observed a major organization of chondrocytes in columns when compared to the YEO group.

### 3.5. YEO Treatment Reduces Paw Edema and Mechanical Hyperalgesia in a CFA-Induced Persistent Inflammation Model

**Figure 6:**
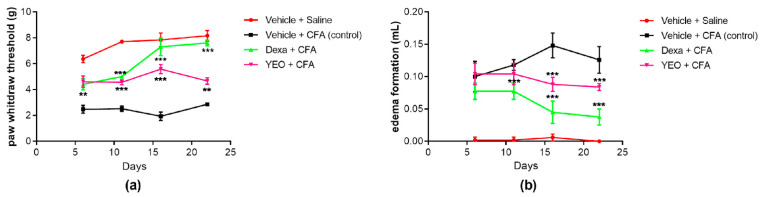
Effects of chronic oral administration of YEO (200 mg/kg) on CFA-induced mechanical hyperalgesia (**a**) and edema formation (**b**) 6, 11, 16, and 21 days after CFA injection. The animals received a single oral administration of YEO (200 mg/kg) once a day for 21 days in the CFA model. Each bar represents the mean ± SEM of 4 animals. ** *p* < 0.01, *** *p* < 0.001, compared with the control group. A two-way ANOVA was performed, followed by the Newman–Keuls test.

The YEO treatment (200 mg/kg) reduced edema formation and mechanical hyperalgesia after CFA-induced persistent inflammation. The effects of YEO were observed in mechanical hyperalgesia and paw edema over 21 days at the time points of 6, 11, 16, and 21 days in this model. On day 16, we observed the highest peak of hyperalgesia (Figure 6a) and edema formation (Figure 6b), compared with the naive group. The YEO treatment at a dose of 200 mg/kg decreased the mechanical hyperalgesia on day 11 (reduction of 83.87%), and on days 16 and 21, the YEO treatment blocked the development of mechanical hyperalgesia induced by CFA. As expected, the dexamethasone decreased mechanical hyperalgesia at all time points tested (Figure 6a). The YEO and dexamethasone treatments did not reduce edema formation on day 6, and on day 11, only dexamethasone reduced the edema formation. On days 16 and 21, the YEO treatment reduced the edema formation by 10 and 20%, respectively, and dexamethasone treatment reduced edema formation by 60 and 70%, respectively (Figure 6b).

### 3.6. YEO Treatment Reduces Nociception in an Acetic Acid-Induced Abdominal Writhing Model

**Figure 7:**
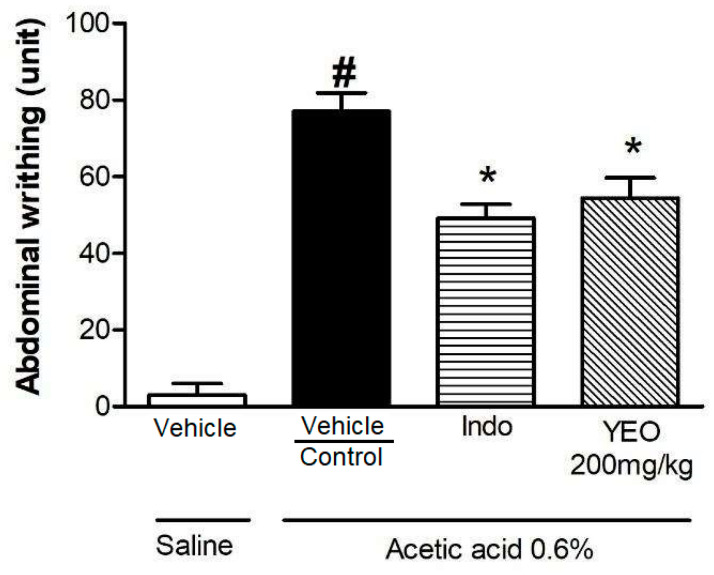
Effect of YEO treatment on acetic acid-induced abdominal writhing. Animals were pretreated 60 min before the challenge, p.o., with the YEO (200 mg/kg), and the animals in the control group received water or indomethacin (n = 5–7 animals/group). Mice were stimulated with an injection via i.p. of 0.6% acetic acid (10 mL/kg), and the number of writhing contortions was recorded over 30 min. Results are expressed as the mean ± SEM. ^#^ *p* < 0.05 compared to saline treatment (vehicle); * *p* < 0.05 compared to the control group (ANOVA, Newman–Keul test).

In a test of abdominal writhing induced by acetic acid, we evaluated the antinociceptive activity of YEO. The acetic acid injection promoted an increase in abdominal writhing (77.00 ± 4.88) in animals that received the vehicle, compared to the saline group. The indomethacin (reference drug) treatment significantly promoted a reduction in writhing (49.20 ± 3.59, reduction of 36.10%) compared to the control group. The YEO treatment (200 mg/kg) significantly reduced acetic acid-induced writhing by 29.35% (54.4 ± 5.34 writhing), compared to the control group (Figure 7).

### 3.7. YEO Treatment Reduces Formalin-Induced Paw Nociception in Mice

**Figure 8:**
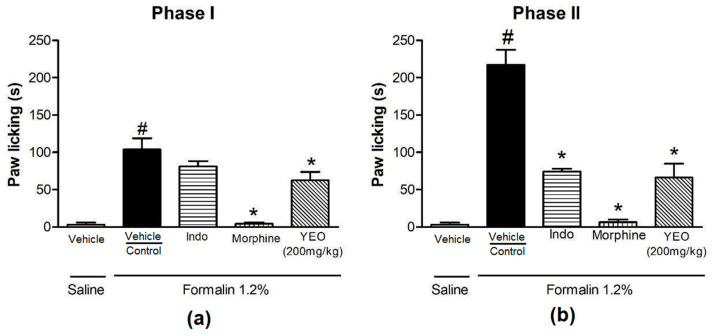
Effect of YEO treatment (200 mg/kg) on formalin-induced paw-licking time. Animals were pretreated 60 min before the challenge, p.o., with YEO (200 mg/kg), and the animals in the control group received water, indomethacin, or morphine. Paw-licking time was timed in the 1st phase (0–5 min) (**a**) and the 2nd phase (15–30 min) (**b**) after intraplantar injections of 1.2% formalin in the hind paws of the mice. Results are expressed as the mean ± SEM. ^#^ *p* < 0.05 compared to saline treatment (vehicle); * *p* < 0.05 compared to the control group (ANOVA, Newman–Keuls test).

The paw-licking time was 103.8 ± 15.18 s in phase I (Figure 8a) and 217.3 ± 20.12 s in phase II (Figure 8b) in the control animals. Oral administration of YEO reduced the paw-licking time in both phases. In phase I, YEO at a dose of 200 mg/kg reduced the paw-licking time by 39.88% compared to the control group (Figure 8a). In phase II, YEO treatment reduced the paw-licking time by 69.44% (66.40 ± 18.22 s) compared to the control group (Figure 8b). In the morphine group, the paw-licking time of the animals reduced by 95.95% (4.20 ± 1.98 s) and 96.96% (6.60 ± 3.44 s) in phase I and phase II, respectively. In the indomethacin group, the paw-licking time was inhibited only in phase II, by 66.99% (71.73 ± 3.91 s).

## 4. Discussion

The results obtained show that YEO exhibited anti-inflammatory, antiarthritic, antinociceptive, and anti-hyperalgesic action in the experimental models. Recently, another study carried out by our research group demonstrated that YEO presented activity on the acute inflammatory response in in vitro and in vivo experimental models, and such activities may be related to the presence of the major constituents found in this essential oil, such as benzyl acetate (18.21%), linalool (15.23%), and benzyl benzoate (11.39%) [14]. Several studies have demonstrated the effects of natural products, essential oils, and terpenes in an experimental arthritis model [21,22]. However, this is the first work that demonstrates the effects of YEO in a model of experimental arthritis and persistent inflammation.

Zymosan, an insoluble polysaccharide derived from the cell wall of the yeast *Saccharomyces cerevisiae* [23], is commonly used to induce an inflammatory response in mice. It is an agonist of Toll-like receptors (TLRs) present on the cell surface, such as TLR-1, TLR-2, and TLR-6, and through this, it induces an activation of the NF-κB transcription factor [24,25]. The zymosan-induced arthritis model is characterized by the release of pro-inflammatory cytokines, such as tumor necrosis factor (TNF), interleukin-18 (IL-18), interleukin-1β (IL-1β), and interleukin-6 (IL-6); synovial joint hypertrophy; and leukocyte recruitment. These inflammatory conditions are similarly observed in RA [26].

Therefore, in this model, zymosan is administered intra-articularlly in the mouse knee, promoting hyperalgesia, joint swelling, leukocyte recruitment, and histopathological changes, lesions similar to those found in RA [27,28,29]. For this reason, it is considered a suitable animal model to study new therapeutic approaches for RA treatment [30]. This model allows the reproduction of lesions to begin within 6 h, initially developing as an increase in vascular permeability, causing local edema and an influx of leukocytes [31], and these cells, when arriving at the injury site, contribute to the lesions with joint damage [32]. Subsequently, 7 days after the induction of arthritis, progressive synovitis occurs with synovial hyperplasia, mononuclear cell influx, fibroblast activation, and articular cartilage and subchondral bone degradation, similar to the chronic synovitis that characterizes the rheumatoid *pannus* [33].

Our results show that treatment with YEO at doses of 100 and 200 mg/kg reduced the infiltration of leukocytes into the joint cavity at the time points of 6 h and 7 days after arthritis induction. Corroborating our results, treatment with YEO was found to reduce leukocyte recruitment in a zymosan-induced peritonitis model, and this effect was related to reduced nitric oxide production [14]. We also demonstrate that the YEO treatment reduced IL-6 levels in the synovial fluid of the mice submitted to zymosan-induced arthritis. It is probable that the reduction in the production of this pro-inflammatory cytokine contributes to the anti-inflammatory activity of YEO. One study showed that linalool, present in the YEO composition, presented in vitro anti-inflammatory activity in macrophages; this terpene promotes reductions in NF-κB and COX-2 expression, as well as prostaglandin production [34]. Huo et al. (2013) [35] demonstrated that linalool inhibits the expression of TNF and IL-6 in LPS-stimulated macrophages. Therefore, the presence of linalool in the YEO composition may be related to the reduction in leukocyte recruitment observed in our study. In addition to the influx of cells into the joint cavity, zymosan-induced arthritis also promotes the formation of joint edema and nociception due to tissue compression in the nociceptive fibers, which, in the presence of pro-inflammatory mediators, such as prostaglandins, can cause hyperalgesia [36]. YEO treatment inhibited edema formation and mechanical hyperalgesia in animals submitted to zymosan-induced arthritis. YEO treatment at doses of 100 and 200 mg/kg was able to reduce the formation of edema, but only at a dose of 200 mg/kg did YEO treatment significantly reduce hyperalgesia in the arthritic mice. For this reason, the dose of 200 mg/kg was utilized for our evaluation of the YEO’s effects on histological changes, CFA-induced persistent inflammation, and nociception models.

To verify changes in articular cartilage, histological analysis was performed 7 days after zymosan-induced arthritis. We observed that the experimental arthritis promoted surface discontinuity, matrix destruction, proliferation, and chondrocyte hypertrophy, causing cartilage destruction, a characteristic present in patients with RA. Our results show that daily treatment with YEO for 7 days was able to reduce the influx of leukocytes into the joint cavity and reduced the cartilage damage after the induction of arthritis, which is evidenced in the cell count and histological analysis results.

CFA is widely used to induce experimental inflammation. This agent produces polyarthritis and persistent inflammation. In this model, the five cardinal signs of inflammation (hypersensitivity, redness, swelling, heat, and loss of function) are observed. The CFA injection promotes chronic inflammation, with a predominance of mononuclear cells [37]. When stimulating inflammatory agents, it is observed that spleen and thymus swellings occur because of T-cell activation and proliferation. This T-cell hyperactivation and infiltration promotes the production of pro-inflammatory cytokines, resulting in a increased inflammatory process, which is a major contributor to the development of inflammatory and autoimmune diseases [38]. Zaringhalam et al. (2008) [39] showed that the inflammation induced by CFA is a biphasic response, wherein the first phase (acute phase) is associated with increased pain and hyperalgesia due to the production of pro-inflammatory cytokines (such as TNF and IL-1β) and COX-2 expression [7], while in the second phase (chronic phase), the hyperalgesia decreases substantially compared to previous days due to the presence of opioid receptors [39]. Thus, the difference between the peaks in hyperalgesia and edema is explained. The maximum inhibition of hyperalgesia promoted by YEO treatment was observed on day 16 and that of edema formation was observed on day 21 after CFA injection.

To evaluate the antinociceptive activity of YEO, experimental models of abdominal writhing induced by acetic acid and paw-licking induced by formalin were used. Pain is the predominant problem in patients with RA. RA pain may be due to joint inflammation and also augmented by central sensitization and joint destruction, and treatment should aim to both suppress the inflammatory disease and relieve pain symptoms [40]. Acetic acid, when administered into the peritoneal cavity, promotes abdominal writhing through the activation of primary afferent nociceptors [41] and the production of inflammatory mediators, such as prostaglandins (PGE_2_, PGF_2α_), vasoactive amines (serotonin and bradykinin) [42], and pro-inflammatory cytokines, promoting a nociceptive response [43]. We found that YEO treatment significantly reduced the number of acetic acid-induced abdominal writhing contortions.

The injection of formalin into a mouse paw results in a reproducible biphasic response that can lead to an inflammatory reaction by activating the release and production of various inflammatory mediators [44]. Phase I (0–5 min) occurs through direct nociceptor sensitization by the noxious stimulus, and phase II (15–30 min) occurs through the production of inflammatory mediators, including prostaglandins, that reduce the threshold for pain, causing inflammatory pain [45]. In the present study, the YEO treatment reduced phase I and phase II in the formalin test, thus proving YEO’s antinociceptive and anti-hyperalgesic activity. Corroborating the results obtained here with YEO treatment in the formalin phase I test, Borgonetti et al. (2022) [17] also demonstrated that YEO reduces neuropathic pain.

## 5. Conclusions

YEO reduced the inflammatory parameters and showed antinociceptive and anti-hyperalgesic activity in mice submitted to zymosan-induced arthritis. YEO treatment also reduced the cartilage damage in the arthritic mice. The anti-inflammatory mechanism of YEO is involved, in part, with a reduction in IL-6 production. YEO can attenuate inflammatory parameters in models of persistent inflammatory diseases, such as RA.

## Figures and Tables

**Table 1:** Cartilage histopathology degradation assessment methodology (adapted from Pritzker et al. [19]).

Grade	Key Features	Associated Criteria (Tissue Reaction)
0	Surface and cartilage morphology are intact	Matrix: normal architecture. Cells: intact, appropriate orientation.
1	Surface is intact	Matrix: superficial cartilage intact, little edema and/or fibrillation (abrasion), focal superficial matrix condensation. Cells: proliferation (clusters) and hypertrophy.
2	Surface exhibits discontinuity	Matrix: superficial discontinuity (erosion). Deep fibrillation near the bone. Cells: disorientation of chondrocyte columns, death, proliferation (clusters), and hypertrophy of chondrocytes.

## Data Availability

All data are available in this publication.
